# Transforming Fundus Photography for Deep Learning-Based Anemia Screening

**DOI:** 10.3390/jcm15145702

**Published:** 2026-07-21

**Authors:** Taeseen Kang, Kiyup Nam

**Affiliations:** 1 Nuri Eye Hospital, Daejeon 35233, Republic of Korea; 2 Department of Ophthalmology, Chungnam National University Hospital, #282, Munhwa-ro, Jung-gu, Daejeon 35015, Republic of Korea

**Keywords:** anemia, deep neural network, fundus photography, hemoglobin as pulse oximetry, near-infrared spectroscopy, transcutaneous hemoglobin monitoring

## Abstract

**Background/Objectives:** This study aimed to develop and evaluate a noninvasive anemia screening method using fundus photographs analyzed by deep learning models, by transforming circular fundus photographs into square images suitable for convolutional neural network analysis. **Methods:** A total of 39,036 fundus images and clinical data collected from 2011 to 2023 were used. Eligible patients had clearly visible macula and optic discs and corresponding hemoglobin measurements. Three image preprocessing methods were applied—untransformed, stretching, and remapping. **Results:** Among preprocessing methods, the stretching method yielded the most accurate predictions. Deep learning analysis was conducted using multi-tasked cascaded convolutional neural networks. The hemoglobin prediction model based on EfficientNet B5 with remapping preprocessing achieved good performance (R^2^ = 0.781, MAE = 1.23), while the anemia classification model demonstrated screening accuracy with AUC values exceeding 0.893. **Conclusions:** Deep learning analysis of fundus photographs demonstrates potential as a noninvasive screening method for anemia. This approach may be particularly beneficial for patients requiring regular hemoglobin monitoring.

## 1. Introduction

Anemia, a common blood disorder characterized by a deficiency in the number or quality of red blood cells (RBCs), affects a significant proportion of the population. The incidence of anemia varies widely, depending on age, sex, geographical location, and socioeconomic status. According to the World Health Organization (WHO), anemia affected approximately 1.92 billion people worldwide in 2021 [1] and resulted in 52.1 million years lived with disability (YLDs) (34.8–75.5), ranking as the leading cause of disability among all health impairments [2,3]. The diagnosis of anemia typically involves a comprehensive approach that includes thorough clinical evaluation and various laboratory tests. The primary diagnostic tool is the complete blood count (CBC), which measures the concentration of hemoglobin, and the number of red blood cells (RBCs), which is traditionally measured using a venous or capillary blood sample [4].

Noninvasive detection methods for anemia, such as photoplethysmography, are gaining increasing attention because of their potential to provide a rapid, painless, and convenient assessment of anemia, specifically in settings where traditional blood tests are not feasible. These methods leverage various technologies to estimate hemoglobin levels or related parameters without the need for venipuncture [5,6,7].

Anemia can manifest various ocular symptoms. One of the most noticeable signs in the eyes is paleness of the conjunctiva, which is a thin membrane lining the front of the eye. This paleness occurs because of reduced hemoglobin levels, leading to insufficient oxygen supply to the conjunctival blood vessels. While the conjunctiva typically presents with a reddish hue owing to its rich vascularization, anemic conditions can significantly diminish this coloration. In addition to conjunctival pallor, individuals with anemia often experience a general sense of fatigue that can affect their eyes.

One effective yet straightforward method for diagnosing anemia is the examination of the conjunctival color. Physicians can gain initial insights into the anemic status of a patient by observing the color of the conjunctiva for any signs of pallor. This approach underscores the significance of a holistic clinical examination, in which even subtle changes in the eye can reveal important information about the overall health of a patient [8]. However, retinopathy did not significantly increase in patients with anemia, and standard fundus photographs are not effective in detecting anemia [9].

Previous studies have reported that deep learning (DL) algorithms utilizing fundus photographs can detect conditions beyond ocular health, such as age, sex, and cardiovascular risk [10,11,12,13,14,15,16,17,18]. In this study, we devised a method to transform circular fundus photographs into square images suitable for deep neural network analysis and focus on accurately predicting hemoglobin levels and screening for anemia using fundus photographs coupled with DL techniques. Through this approach, we aim to explore methods for effective screening of anemia using fundus photographs.

### Background

Blood is supplied to the eye via the central retinal artery and posterior ciliary artery; specifically, the central retinal artery supplies the retina through the optic disc, while the posterior ciliary artery supplies the choroid. In anemia screening, the stable blood flow within the choroid and the optic disc serves as a critical biological marker, as the color density and pallor of these structures vary according to hemoglobin concentration. Deep convolutional neural networks can extract these spatial and color patterns to enable noninvasive hemoglobin quantification.

Deep neural networks, particularly convolutional neural networks, have demonstrated remarkable efficiency in processing and analyzing highly standardized medical imaging modalities. Standardized formats like digital fundus photography provide consistent anatomical landmarks—such as the optic disc, macula, and major vascular arches—within a uniform frame. This spatial and structural consistency allows deep learning models to minimize the noise typically found in heterogeneous imaging data, accelerating the feature-extraction process and enabling high-throughput analysis with exceptional precision [19].

Gradient-weighted Class Activation Mapping (GradCAM) was developed to explain CNN decisions [20]. It utilizes the gradient information flowing into the final convolutional layer to highlight positive feature importance. The resulting localization heatmap is superimposed onto the original image, with redder hues representing the areas of maximum importance to the network.

## 2. Methods

### 2.1. Datasets

Fundus photographs and demographics were obtained between October 2011 and August 2023. The inclusion criteria were cases in which both hemoglobin and fundus photographs were taken on the same day and where the macula and optic disc could be observed in the fundus photographs. The exclusion criteria included cases in which the image quality was poor and the macula and optic disc were difficult to discern. Patients were excluded if data on blood hemoglobin concentration, age, or sex were unavailable.

The fundus photographs were taken in an ophthalmology outpatient clinic by multiple examiners using two Canon CR-1 fundus cameras (Canon Inc., Tokyo, Japan). A convolutional neural network model was trained to classify the suitability of fundus photographs according to the inclusion criteria. The classified images were subsequently reviewed and verified by a single ophthalmology specialist.

**Figure 1:**
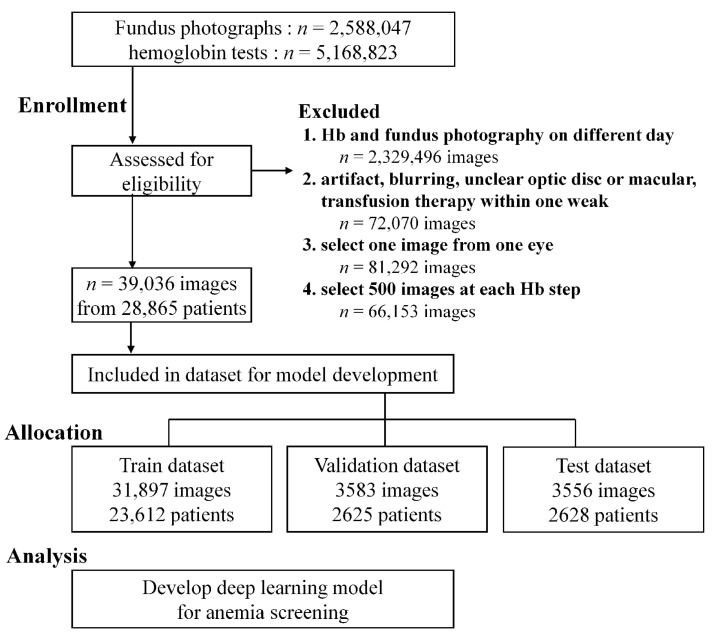
Study profile of dataset selection.

**Figure 2:**
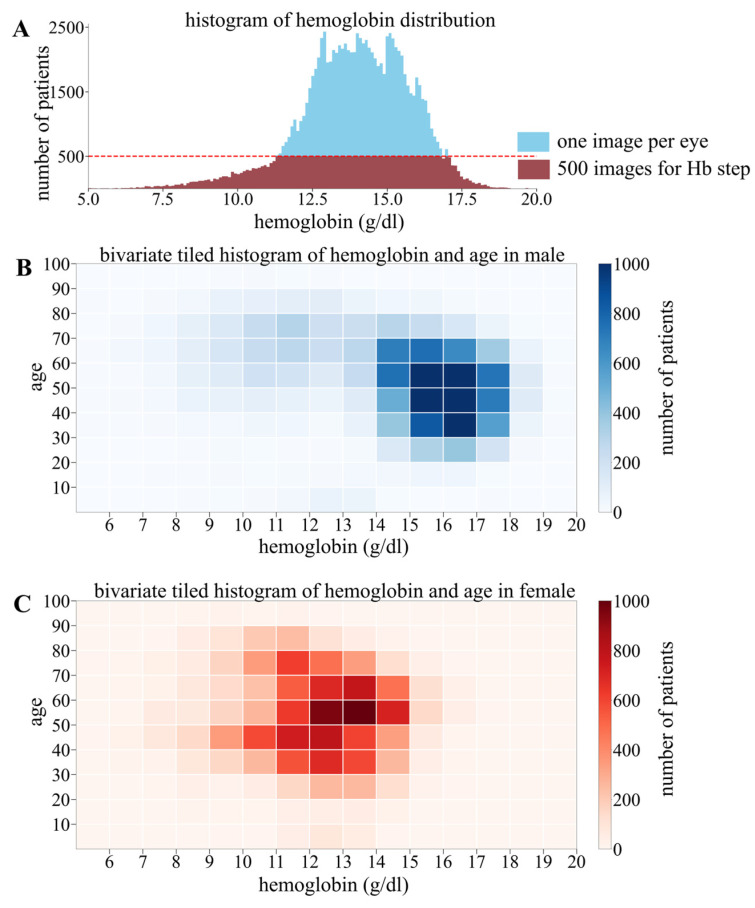
Distribution of hemoglobin concentration and age. (**A**) Histogram of hemoglobin distribution before sampling (sky-blue) and after data balancing (brown), where a maximum of 500 images were randomly selected via simple random sampling for each 0.1 g/dL bin (red dashed line). (**B**,**C**) Bivariate tiled histograms for male and female groups illustrating the relationship between hemoglobin concentration (1 g/dL bins) and age (10-year bins), with color intensity reflecting image density.

The process of dataset selection is shown in Figure 1. During the process, 2,588,047 fundus photographs and 5,168,823 clinical data points were obtained. From 53,848 patients, 186,481 fundus photographs met the inclusion criteria. To ensure data independence, only one image was used per eye. To reduce the imbalance in the hemoglobin data distribution, we randomly selected 500 fundus photographs for each 0.1 g/dL interval of hemoglobin, without adjusting for age, sex, or eye laterality. When multiple hemoglobin tests were conducted within a single day, only the initial test result was included in the analysis. If a hemoglobin interval contained fewer than 500 fundus photographs, all available photographs were included (Figure 2A). Finally, 39,036 fundus photographs from 28,865 patients were included. Of these, 31,897 images (81.7%) were used for the training, 3583 images (9.2%) for the validation dataset, and 3556 images (9.1%) were used for the test dataset. We performed a patient-level split during dataset creation to minimize the risk of information leakage. The number of cohorts by Hb bin can be provided upon request.

The distribution of age, sex, and hemoglobin data used in this study is presented in Figure 2. Our dataset shows age ranges from 0 to 100 years, and hemoglobin values range from 5 to 25 g/dL. The hemoglobin levels of males were higher than those of females included in this study.

### 2.2. Fundus Photography

We retrieved fundus photographs from the PACS and saved them in the PNG file format. This study focused on the central area of fundus. Therefore, we excluded fundus photographs in which the optic disc and macula could not be identified simultaneously. Only one image per eye was used to reduce overfitting. In instances where multiple fundus photographs were obtained from the same eye, only the most recently captured images were selected. The fundus photographs were trimmed to remove the peripheral black blank areas.

### 2.3. Preprocessing: Fundus Photographs Transformation Method

**Figure 3:**
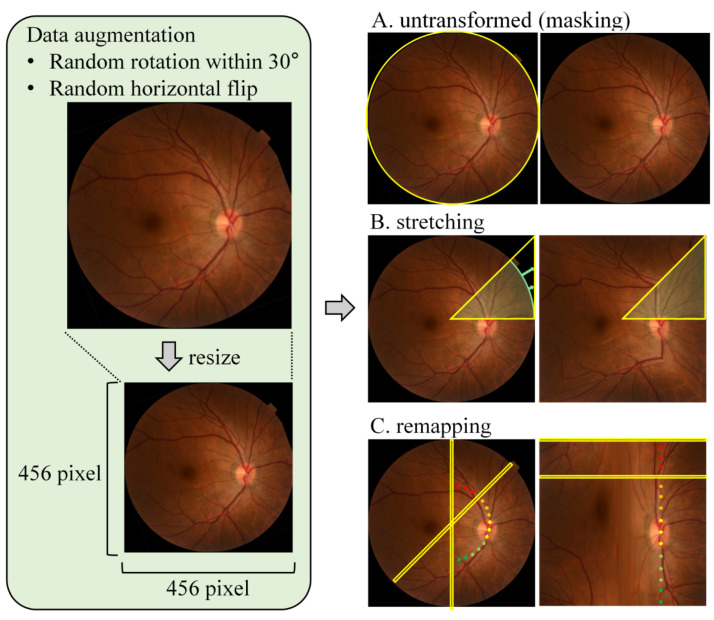
Fundus photograph preprocessing methods. Original images underwent data augmentation via random horizontal flips and random rotations, followed by resizing. The stretching method converts the circular image into a rectangle using eight triangularly reshaped sectors, while the remapping method achieves rectangular reprojection by shifting pixels based on radial coordinates and angles. (**A**) untransformed with standard circular masking; (**B**) stretching, where cyan arrows indicate radial expansion into the corners; and (**C**) remapping, where colored dots show how a curved region is realigned vertically.

We presented two transformation methods for reshaping circular fundus photographs into rectangular shapes to enhance the performance of artificial intelligence. The first method is stretching transformation, in which a circular fundus photograph is divided into eight sections to create a fan shape with a central angle of 45°. Each fan shape is transformed into a right triangle. The triangles on the right are combined to form a rectangular image (Figure 3A). The second method is a remapping transformation, which involves acquiring the diameter of a circle by rotating it clockwise and then combining them. For each point in the fundus photograph, a new coordinate system was created using the distance and angle from the center, and remapping was performed using the grid sample function of PyTorch version 3.10.9 (Figure 3B). This study compared the outcomes of three different methods: untransformed, stretching, and remapping. The detailed Python version 3.10.9 code for the transformation methods is provided in the Supplementary Material.

In this study, the entire preprocessing process was as follows: The photographs were resized to a 456 × 456 pixel image. A random rotation of 30°and random horizontal flips were performed for data augmentation. Subsequently, stretching or remapping was performed, if necessary. The images were normalized by dividing by 255.0 to ensure that the values ranged between 0 and 1.

### 2.4. Deep Neural Networks Model: EfficientNet B5 and Anemia Screening Model

**Figure 4:**
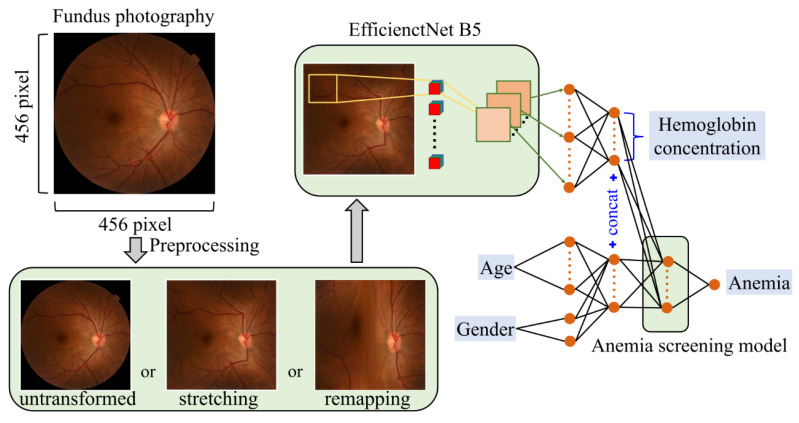
Schematic of the multitask cascaded CNN architecture. The framework consists of an EfficientNet-B5 backbone for predicting hemoglobin concentration, followed by an anemia screening model that integrates demographic data. Three independent pipelines evaluate different preprocessing methods: untransformed, stretching, and remapping. The arrow indicates data flow to the feature extractor, and orange circles denote fully connected nodes.

Three independent deep neural network models were developed, each trained using a different preprocessing method (Figure 4). The models consisted of a multitask cascaded convolutional neural network (CNN). The first component is responsible for predicting the hemoglobin concentration. The second component, an anemia screening model, assesses the presence or absence of anemia by incorporating demographic information.

To accurately predict hemoglobin levels from fundus photographs using a deep neural network, we used the EfficientNet B5 model [21]. The trainable variables were initialized using a Gaussian distribution. The key hyperparameters for model training included a batch size of 20,100 epochs, and a learning rate set at 0.002. The model was optimized using the Adam optimizer. Binary Cross-Entropy with Logits Loss (BCEWithLogitsLoss), for enhanced numerical stability, was employed as the loss function. The EfficientNet-B5 model outputs a 150-dimensional vector representing discrete hemoglobin levels ranging from 5.0 to 20.0 g/dL at 0.1 g/dL intervals. This output is subsequently concatenated with an 8-bit binary-coded age feature and a 1-bit sex indicator to form a 159-dimensional feature vector. This integrated vector is then fed into a secondary deep neural network to predict the presence of anemia. The hemoglobin level is determined by applying a softmax function to the EfficientNet-B5 outputs and selecting the index with the highest probability. The specific hyperparameters are presented in Table 1.

Anemia screening models that can determine the presence of anemia using the output of EfficientNet B5 were developed. The model was implemented as a deep neural network that uses the output of EfficientNet B5, along with age and sex, as inputs to determine the presence of anemia. It is composed of 12 fully connected layers. In this study, the diagnostic criteria for anemia were based on WHO standards [22], defining anemia as hemoglobin levels below 13 g/dL for men and below 12 g/dL for women.

Three independent models were developed, and each model was trained using a different preprocessing method: untransformed, stretching transformation, and remapping transformation.

### 2.5. Statistical Analysis

We qualitatively evaluated the variables using the class activation map (CAM) method [20,23]. GradCAM was employed to visualize the decision-making processes of the model, producing heatmaps that highlighted areas critical to classification by the CNN. The areas with redder hues signify higher importance and play a crucial role in the CNN class discrimination process. The CAM was used to pinpoint areas of significance within the final convolutional and classification layers of the network. This approach validates the appropriateness of the model and identifies the key regions in fundus photographs that are critical for generating outputs [20]. Guided-back propagation was used to identify regions that positively contributed to the prediction by tracking gradients. This method focuses on positive gradients, ignores the negative gradients within each layer of the algorithm, and traces them back to specific locations on the input images that are positively associated with the prediction outcome [23].

EfficientNet B5 was used to predict the hemoglobin concentration. The predicted and measured hemoglobin concentrations were assessed by plotting a scatter plot and evaluating the slope and correlation coefficient. Additionally, a Bland–Altman plot was created to compare the error ranges. An ROC curve was constructed for the anemia screening model, and the area under the curve (AUC) was calculated to evaluate the performance of the model.

Statistical analyses were performed using Python software version 3.10.9, PyTorch 2.0.0, NumPy 1.23.5, and OpenCV-Python 4.7.0.72. The performance of the CNN model was evaluated on the basis of the accuracy of the test set. The CNN model was trained using an Intel^®^ Core™ i9-10980XE CPU (Intel Corporation, Santa Clara, CA, USA) and an NVIDIA^®^ GeForce RTX 6000 Ada graphics card (NVIDIA Corporation, Santa Clara, CA, USA).

## 3. Results

The demographic information of the data used in this study is presented in Table 2. Of the total data, 30.2% of patients met the criteria for anemia. The average age was 52 years and the average hemoglobin concentration was 13.6 g/dL. When comparing the training, validation and testing sets, there were no significant differences in age, sex, hemoglobin levels, or proportion of anemia.

**Figure 5:**
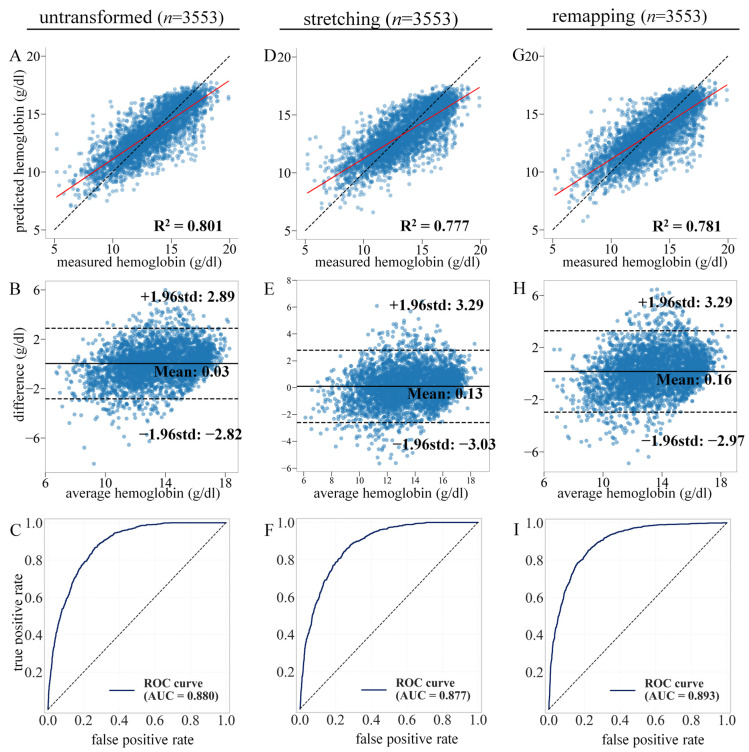
Model performances for hemoglobin prediction using EfficientNet B5 and anemia screening model. Results for the untransformed (**A**–**C**), stretching (**D**–**F**), and remapping (**G**–**I**) methods are shown. (**A**,**D**,**G**) are scatter plots that display the relationship between measured and predicted hemoglobin concentrations from EfficientNet B5. Each dot represents the predicted value against the measured value, with the black line indicating the ideal model prediction and red solid lines indicate the line of linear regression. (**B**,**E**,**H**) are the Bland–Altman plots illustrating the differences between predicted and measured values plotted against the average of the measured and predicted values. The black solid line shows the mean difference, and the dashed black lines represent the 95% limits of agreement. (**C**,**F**,**I**) display the ROC curves for the anemia screening model, evaluating the ability of the model to distinguish between patients with and without anemia.

Three independent models were developed, each trained using different preprocessing methods. The performances of these models are summarized in Figure 5. Figure 5A–C present the results from models using untransformed images. Figure 5D–F present the outcomes from models trained with images stretched from circular to rectangular shapes. Figure 5G–I present results from models using remapped images. Scatter diagrams of predicted versus measured hemoglobin concentrations are shown in Figure 5A,D,G, while the Bland–Altman plots for these predictions are shown in Figure 5B,E,H.

The results of hemoglobin estimation using the fundus photographs and EfficientNet B5 were excellent across all models. In the scatter plots, all models showed results that were well-clustered around the centerline. The R^2^ values for the models using the untransformed, stretching, and remapping pre-processing methods were 0.834, 0.777, and 0.781, respectively. The mean absolute errors (MAE) of the models were 1.19, 1.24, and 1.23, respectively. This demonstrates excellent performance across all models, with the remapping method slightly outperforming the other methods. The slope values of the scatter plots were 0.801 for the untransformed method, 0.978 for the stretching method, and 0.945 for the remapping method. These results indicate that the stretching method with a slope closer to 1 exhibited superior performance. In the Bland–Altman plots, the intervals between the upper and lower limits of agreement were 5.71, 6.32, 6.26 for the untransformed, stretching, and remapping methods, respectively, further indicating that the stretching method achieved the highest level of agreement.

The performance of the anemia screening model utilizing hemoglobin predictions from EfficientNet B5 and demographic information is displayed in Figure 5C,E,I via the ROC curves. The AUC values for the untransformed, stretching, and remapping methods were 0.882, 0.877, and 0.893, respectively. This indicates similar performances for the untransformed and stretching methods, whereas the remapping method showed a slightly inferior performance.

An ablation study was conducted to cautiously evaluate the respective impacts of input modalities and transformation techniques. Utilizing only fundus images for anemia prediction yielded a baseline AUC of 0.717. When age and sex variables were integrated without data augmentation, the AUC rose to 0.846. Meanwhile, the untransformed method proposed in this study achieved an AUC of 0.882, and the remapping method reached 0.893. These steady increments suggest that our preprocessing approaches may contribute to incremental improvement in overall predictive performance.

**Figure 6:**
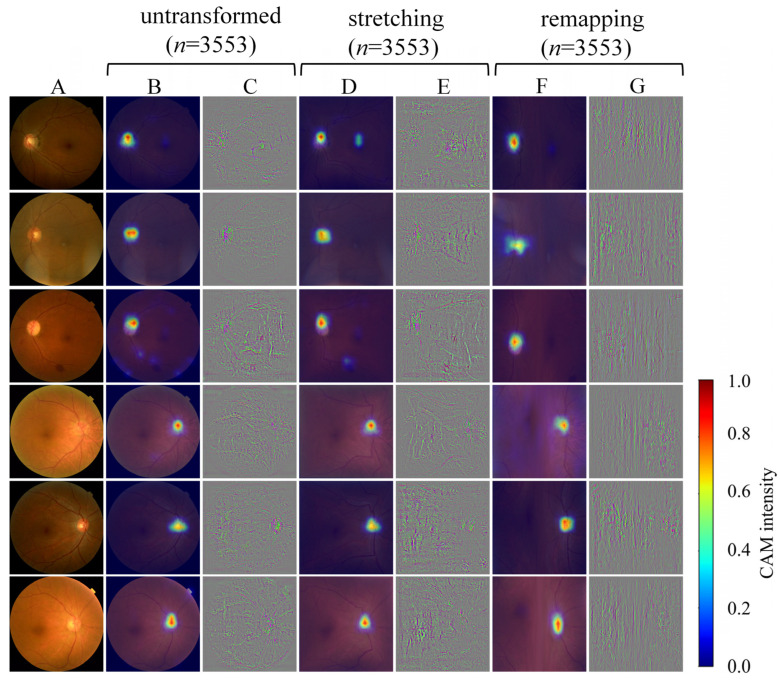
Examples of applying GradCAM and guided backpropagation to highlight the region’s model while focusing on the prediction. (**A**) Original fundus image. (**B**,**C**) Results from GradCAM and guided backpropagation of the untransformed fundus photograph, highlighting the areas of interest. (**D**,**E**) GradCAM and guided backpropagation results for the stretched fundus photograph, indicating the focused regions. (**F**,**G**) Results from GradCAM and guided back propagation for the remapping fundus photograph showing the areas on which the model concentrates.

Figure 6 illustrates the results using GradCAM and guided backpropagation techniques. All the models emphasized the optic disc. The untransformed and stretching methods also highlight the retinal vessels, with some focus on the macular area. However, in the remapping method, retinal vessels were not distinctly distinguished.

## 4. Discussion

Fundus photography can be used as a biomarker in medical evaluations to assess systemic conditions [24,25,26]. The eye is the only organ that allows direct visualization of blood vessels in the body [27] and the retina is rich in arteries, veins, capillaries, and nerve fibers. Therefore, retinal changes are closely related to cardiovascular and neurological diseases [28]. Fundus photography is a quick and painless test that defines the examination and method for evaluating blood vessels and nerves as a health checkup [29].

Several studies have investigated anemia prediction using fundus photographs. A study utilizing the UK Biobank dataset reported an MAE of 0.63 g/dL [16], while a cohort study conducted in southern India reported an MAE of 0.58 g/dL [30]. Image-based approaches using body regions other than the fundus have also been reported. In this study, predicting anemia using remapping fundus photographs resulted in an AUC of 0.893, which is similar to or slightly improved from the 0.86–0.88 reported in studies using macular images [15,16]. The MAE in the model predicting hemoglobin was 1.23 g/dL. (Table 3).

The relatively higher MAE observed in this study can be attributed to the high prevalence of anemia in our dataset. As illustrated in Figure 3, while the model accurately predicts hemoglobin levels within the normal range, the prediction error increases for lower hemoglobin concentrations. Because a substantial portion of the normal-range data was excluded during the balancing process to correct for class imbalance, the relative weight of low-hemoglobin samples became more pronounced, thereby driving up the overall MAE.

The performance of anemia prediction from fundus photographs appears to be influenced by features around the macula and the optic disc. GradCAM imaging identified the optic disc and macula as regions for predicting hemoglobin concentrations, which is consistent with the results of previous studies [15,16]. The optic disc is a zone in which many blood vessels converge. The central retinal artery and vein branch out, supplying the entire retinal area and thereby effectively representing the retinal vessels. The macula, which is the thinnest part of the retina, and the area where the choroid is the thickest, represent the choroidal region. Therefore, the optic disc and macula are crucial for predicting hemoglobin concentrations from fundus photographs.

In the fundus photographs, changes in color indicate the amount of hemoglobin. The choroid of the eye, a rich supply of blood vessels, and a lack of autonomic nervous system maintain constant blood flow. Similarly, blood flow in the optic disc remains stable regardless of blood pressure variations [31]. Areas of the optic disc with high hemoglobin content predominantly reflect red light, whereas areas with low hemoglobin content show a reduced proportion of red light compared with green and blue light [32]. These characteristics enable the accurate measurement of hemoglobin concentrations in fundus photographs.

A novel aspect of this study is the preprocessing of fundus photographs in various ways to enhance the performance of the artificial intelligence system. Fundus photographs were typically circular (Figure 6A), resulting in an information-blank space at the periphery. Because AI systems can only process rectangular images, this peripheral blank space can result in wasted computational resources. Additionally, the blank space at the periphery may negatively affect machine learning. The stretching transformation technique extends the circular fundus photograph to a rectangular configuration at a consistent ratio (Figure 3A). This method offers the advantage of preserving the integrity of the macular and optic disc regions, albeit with the drawback of image distortion at 45°and 135°. The remapping transformation involves computing the diameter of the circle through clockwise rotation and subsequent combination (Figure 3B). However, this method notably distorts the central area of the image, particularly affecting the macular region. The AI anemia model trained with untransformed images demonstrated sufficient accuracy; however, the remapping transformation technique was slightly more precise in predicting the hemoglobin levels. This suggests that the peripheral blank area in the untransformed images has a deleterious effect on the performance of deep neural network, highlighting the impact of noninformative peripheral blanks on learning algorithms. These findings highlight the critical influence of image preprocessing on the performance of neural network models in medical imaging applications.

The strengths of this study include the long-term data collection from a large patient group and multiple operators using various fundus photography equipment at two tertiary hospitals. Additionally, it demonstrated a slight improvement in AI performance through transformation techniques. Our results are nearly identical to those reported in the literature. In addition to EfficientNet, we tested several renowned AI models and obtained nearly consistent results. The CNN structure may have similar limitations in predicting hemoglobin from fundus photographs. Recently, the enhanced performance of the attention mechanisms used in large language models has garnered significant interest, and their application in image analysis is increasing. We plan to conduct further studies using these new mechanisms in conjunction with CNNs.

Using fundus photography to predict anemia is useful in patients with an aversion to traditional blood draws, such as children, the elderly, or those with conditions requiring frequent monitoring. This method could make anemia screening as simple as routine eye examinations, thereby increasing the frequency and reach of screening [33]. In addition, it could be transformative in managing chronic diseases, such as renal failure or cancers, where anemia is a common complication, and early intervention can improve the quality of life. This technology has potential integration with telemedicine platforms, allowing the remote monitoring of patients’ hemoglobin levels without requiring them to visit healthcare facilities [34]. For example, there is a case of using fundus photography to evaluate hemolysis and anemia in astronauts in space [35].

Although this study has several strengths, it also has significant limitations. Due to the lack of records on the examiners and camera devices, these variables were omitted from our analysis. While hemoglobin levels typically undergo a diurnal variation of roughly 0.4 g/dL [36], we were unable to consider this variability as the exact timing of blood sampling was unknown. In addition, due to the restricted scope of our IRB approval, external validation using an independent hospital could not be performed; consequently, the participants were predominantly East Asians from two tertiary hospitals in Korea, which limited their geographical and racial representativeness. Another limitation of this study is the absence of comprehensive clinical variables, such as patient comorbidities and medication histories (such as bleeding disorders, renal disease, iron supplements, or erythropoiesis-stimulating agents). This omission may lead to a discrepancy between our experimental results and actual clinical practice. Other limitations include the lack of standardization across various fundus photography equipment, exclusion of specific patient groups, potential distortions from image preprocessing techniques, reliance on a particular AI model, absence of practical considerations for clinical implementation, and lack of data availability for external validation. These limitations underscore the need for future research to enhance the reliability and applicability of AI models in medical diagnostics, particularly in anemia screening using fundus photography.

## 5. Conclusions

This study demonstrates that novel fundus photograph transformation methods—specifically stretching and remapping—combined with a multitask cascaded deep learning model can effectively detect anemia and quantify hemoglobin levels. By reformatting circular retinal images to minimize noninformative peripheral space, our proposed framework optimized convolutional neural network performance, achieving high screening accuracy with AUC values up to 0.893. These findings highlight the robust potential of automated, fundus-based systems as a noninvasive, rapid, and accurate alternative for anemia screening. This study may improve clinical decision-making in primary care settings and integration into telemedicine platforms, facilitating easier monitoring for high-risk patients.

## 6. Future Works

The image transformation methodologies proposed in this study were specifically designed to adapt circular fundus photographs for deep neural networks that inherently require square inputs. The ‘stretching’ method expands the peripheral and central regions at an identical ratio, thereby preserving anatomical continuity. Conversely, the ‘remapping’ method selectively magnifies the central macular region while compressing the periphery, which compromises overall structural continuity. While the remapping approach may offer distinct advantages for diseases where macular are paramount, the stretching approach is inherently superior when global anatomical structures are critical. Ultimately, integrating both transformation techniques in future deep learning-based fundus studies could synergistically enhance model performance. Future work will focus on integrating both techniques into a multi-branch network to synergistically exploit local and global features. Additionally, we plan to validate our models on diverse, multi-center datasets to ensure broader clinical generalizability.

## Figures and Tables

**Table 1:** Summary of model hyperparameters and training settings.

Category	Hyperparameter	Value
Model training	Initial learning rate (lr)	0.002
	Total training epochs (e)	100
	Batch size	20
Optimization	Optimizer algorithm	Adam
	Loss function criterion	BCEWithLogitsLoss
	Learning rate scheduler	ReduceLROnPlateau
Data features	Hemoglobin output dimensions (number of classes)	150
	Age format	8-bit binary
	Sex format	One-Hot Encoded

**Table 2:** Demographical information.

	Total	Train Set	Validation Set	Test Set	*p*-Value
*n* (%)	39,036 (100.0)	31,897 (81.7)	3583 (9.2)	3556 (9.1)	0.082 *
right eye (*n*)	19,565	15,975	1755	1835	
left eye (*n*)	19,471	15,922	1828	1721	
Age (mean ± SD)	52.12 ± 15.94	51.94 ± 15.94	52.94 ± 15.94	52.44 ± 15.99	0.079 ^†^
Sex					
Male (%)	20,616 (52.8)	16,823 (52.7)	1899 (53.0)	1894 (53.5)	0.817 *
Female (%)	18,420 (47.2)	15,074 (47.3)	1684 (47.0)	1662 (46.5)	
Hemoglobin					
(g/dL mean ± SD)	13.56 ± 2.52	13.56 ± 2.51	13.56 ± 2.54	13.56 ± 2.56	0.865 ^†^
Anemia					
True (%)	11,789 (30.2)	9604 (30.1)	1092 (30.4)	1093 (30.7)	0.690 *
False (%)	27,247 (69.8)	22,293 (69.9)	2491 (69.6)	2463 (69.3)	

* chi-square; ^†^ One-way ANOVA.

**Table 3:** Performance comparison with previous studies for anemia screening.

Study	Imaging Modality	Prevalence of Anemia	Dataset	Hemoglobin Prediction MAE (g/dL)	Anemia Screening AUC
Mitani et al. (2020) [16]	Fundus images	3.5%	UK Biobank (*n* = 114,205)	0.63	0.88
Zhao et al. (2022) [15]	Ultra-wide-field fundus images	15.9%	China (*n* = 11,528)	0.83	0.93
Khan et al. (2025) [30]	Fundus images	17.1%	South Indian (*n* = 2265)	0.58	0.98
Proposed Method (Remapping)	Transformed fundus images	30.2%	Korean (*n* = 39,036)	1.23	0.89

## Data Availability

The datasets generated and analyzed in the current study are not publicly available because they exceed the scope of IRB approval. However, these datasets can be made available from the corresponding author upon reasonable request.

## Supplementary Materials

The following supporting information can be downloaded at: https://doi.org/10.5281/zenodo.21446963, Source Code S1: Python script containing image transformation methods, including rectByStretch and rectByDiameter implementations in both NumPy and PyTorch.
